# Self-assembled particulate vaccine elicits strong immune responses and reduces *Mycobacterium avium* subsp. *paratuberculosis* infection in mice

**DOI:** 10.1038/s41598-020-79407-7

**Published:** 2020-12-18

**Authors:** Sandeep K. Gupta, Natalie A. Parlane, Dongwen Luo, Bernd H. A. Rehm, Axel Heiser, Bryce M. Buddle, D. Neil Wedlock

**Affiliations:** 1 Grasslands Research Centre, AgResearch, Hopkirk Research Institute, Private Bag 11008, Palmerston North, 4442 New Zealand; 2 Bioinformatics and Statistics, AgResearch, Palmerston North, New Zealand; 3 Centre for Cell Factories and Biopolymers, Griffith Institute for Drug Discovery, Griffith University, Brisbane, QLD Australia; 4 Menzies Health Institute Queensland (MHIQ), Griffith University (Gold Coast Campus), Southport, Australia

**Keywords:** Immunology, Vaccines, Protein vaccines

## Abstract

*Mycobacterium avium* subspecies *paratuberculosis* (MAP) causes chronic progressive granulomatous enteritis leading to diarrhoea, weight loss, and eventual death in ruminants. Commercially available vaccines provide only partial protection against MAP infection and can compromise the use of bovine tuberculosis diagnostic tests. Here, we report the development of a protein-particle-based vaccine containing MAP antigens Ag85A^202–347^-SOD^1–72^-Ag85B^173–330^-74F^1–148+669–786^ as a fusion (‘MAP fusion protein particle’). The fusion antigen displayed on protein particles was identified using mass spectrometry. Surface exposure and accessibility of the fusion antigen was confirmed by flow cytometry and ELISA. The MAP fusion protein particle vaccine induced strong antigen-specific T-cell immune responses in mice, as indicated by increased cytokine (IFN-γ and IL-17A) and costimulatory signals (CD40 and CD86) in these animals. Following MAP-challenge, a significant reduction in bacterial burden was observed in multiple organs of the mice vaccinated with the MAP fusion protein particle vaccine compared with the PBS group. The reduction in severity of MAP infection conferred by the MAP fusion protein particle vaccine was similar to that of Silirum and recombinant protein vaccines. Overall, the results provide evidence that MAP antigens can be engineered as a protein particulate vaccine capable of inducing immunity against MAP infection. This utility offers an attractive platform for production of low-cost particulate vaccines against other intracellular pathogens.

## Introduction

*Mycobacterium avium* subspecies *paratuberculosis* (MAP) causes paratuberculosis or Johne’s disease (JD), which is a progressive, chronic, and highly prevalent disease affecting ruminants worldwide^1,2^. Clinically infected animals develop chronic diarrhoea, gradual weight loss, reduced milk production, decreased fertility, often resulting in premature culling or natural death, which accounts for considerable economic losses^3,4^. Moreover, MAP infection has been associated with Crohn’s disease in humans, although, it has not been established that MAP is the cause of this disease^5,6^. The current commercial vaccine licensed for use in cattle, Silirum (Zoetis, NSW, Australia), contains heat-killed MAP and can reduce the level of bacterial shedding in faeces and severity of JD^7^. However, this vaccine does not provide complete protection against the disease and it interferes with the current diagnostic skin test for bovine tuberculosis^8,9^. Therefore, there is an urgent need to develop an effective vaccine that not only provides protection against MAP infection but is compatible with the diagnosis of bovine tuberculosis.

In recent years, the focus on developing vaccines to control JD has shifted towards the use of subunit vaccines, such as native or recombinant protein and DNA-based vaccines^10,11^. Moreover, recombinant protein-based subunit vaccines are often associated with high production costs due to time-consuming purification processes. These limitations necessitate the development of alternative vaccination strategies.

Efficient and targeted delivery of antigens to appropriate immune cells is crucial for developing successful vaccine formulations^12–14^. Over the past decade, advancements have been made in using particulate type vehicles for antigen delivery, which can overcome the limitations of existing vaccines such as poor antigen presentation, and high production costs^10,11^. A wide range of particles have been used to display antigens including virus-like particles, bacteria-based vectors, liposomes, immune-stimulating complexes, inclusion bodies, and biological polyester inclusions^15–17^. Polyhydroxybutyrate (PHB) is a naturally occurring polyester that forms protein-coated inclusions and is produced by a wide range of bacteria and archaea to serve as an energy source during carbon starvation^18^. Multiple chains of these biopolyesters can assemble to form spherical granules (biobead) of ~ 200–500 nm in size, with PHA synthase (PhaC). Bacteria can be bioengineered by introducing genes that encode the three enzymes PhaA, PhaB, and PhaC to enable recombinant PHB synthesis. The desired antigen can be fused to a PhaC to be displayed on the surface of the PHB beads. An alternative to biobeads are protein particles, where the foreign proteins can be displayed in multiple copies onto the surface of insoluble PhaC particles^17^. Protein particles have several advantages over conventional vaccines, such as low production cost and ease of manufacture, and potentially enhanced efficacy resulting from enhanced antigen uptake due to small particle size and co-delivery of multiple antigens on the same particle.

Chandra et al., employed attenuated Salmonella vector to express fusion of MAP antigens Ag85A-SOD-Ag85B and 74F as part of their secretory pathways^19^. However, the authors were only able to express MAP antigens Ag85A^202–347^-SOD^1–72^-Ag85B^173–330^ and 74F^1–148+669–786^ as truncated secretory proteins in two separate Salmonella vectors. The Salmonella vectors expressing the MAP antigens reduced MAP infection in spleen and liver tissues of experimentally challenged mice^19^. Here, we describe a single plasmid-based system to produce protein particles fused to PhaC without PhaA and PhaB precursor enzymes. In this system, we successfully produced protein particles displaying different regions of MAP antigen complex 85 (Ag85), superoxide dismutase (SOD) and a polyprotein (74F) as a single fusion protein in the order Ag85A^202–347^-SOD^1–72^-Ag85B^173–330^-74F^1–148+669–786^ (MAP fusion antigen). We evaluated the immune responses and protective efficacy of the protein particles vaccine in a MAP challenge mouse model. While mice are not natural hosts for MAP and do not exhibit typical clinical signs of MAP infection (diarrhea and weight loss)^20^, they are often used as models in preliminary vaccine efficacy studies because of cost, practicality and availability of immunological reagents. Calves and goats are natural hosts of MAP but the long incubation period of MAP infection to progress to clinical stage makes preliminary experiments with these animals extremely expensive. Therefore, to develop an effective particulate vaccine against JD in ruminants, as an initial step we evaluated the ability of the protein particles vaccine to stimulate the immune responses and to provide protective efficacy in a mouse model.

## Results

### Production and characterization of protein particles displaying MAP fusion antigen and recombinant proteins in *E. coli*

**Figure 1:**
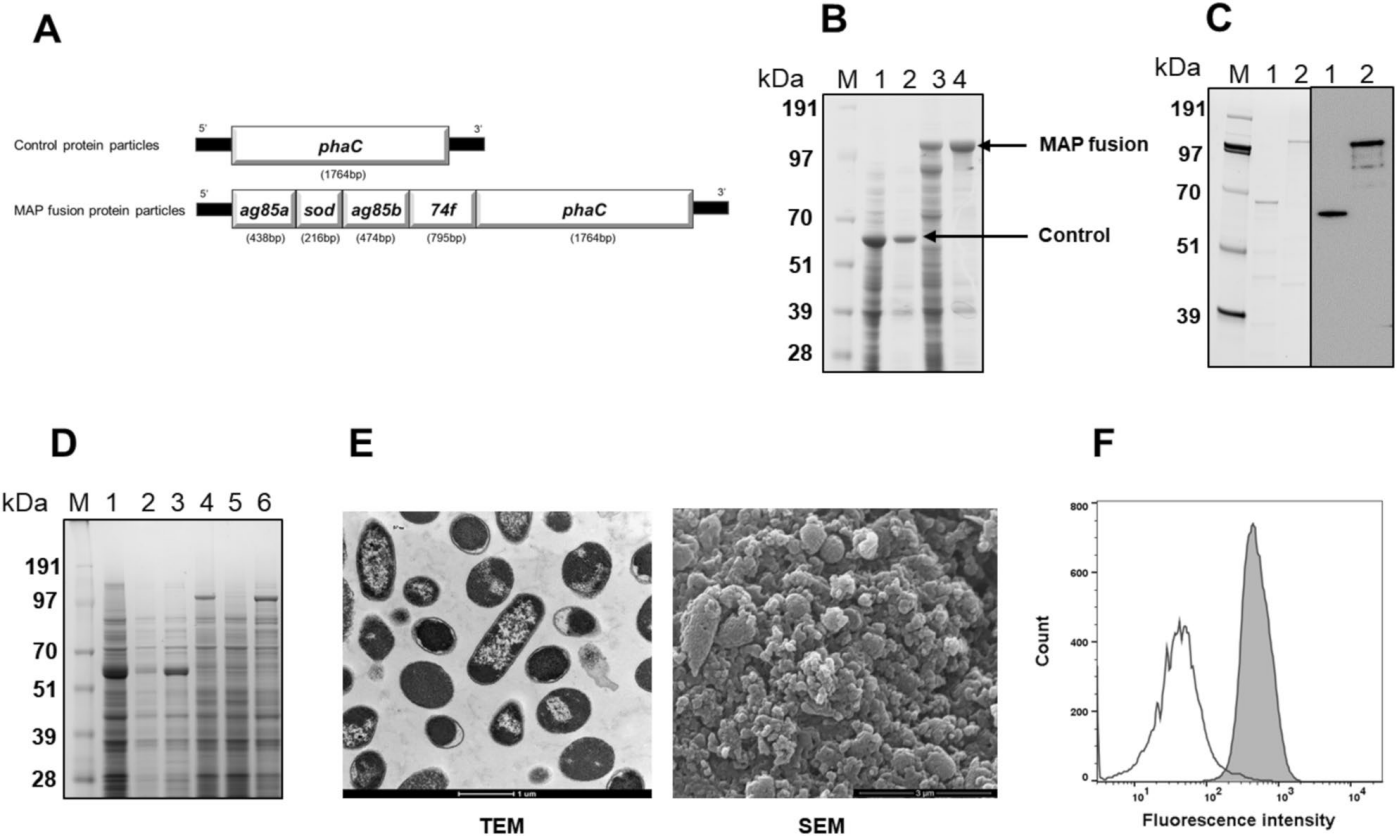
Production and characterization of MAP antigens as protein particles and recombinant proteins in *E. coli*. (**A**) Schematics of genes coding to produce control and MAP fusion protein particles in *E. coli*. (**B**) SDS-PAGE analysis of purified protein particles. *E. coli* cells expressing protein particles were lysed using a microfluidizer and protein particles were purified by centrifugation. Lane M, SeeBlue Plus2 Pre-Stained Standard; lane 1, whole cell lysate from control protein particles producing *E. coli*; lane 2, purified control protein particles; lane 3, whole cell lysate from MAP fusion protein particles producing *E. coli*; lane 4, purified MAP fusion protein particles. A full-length gel image is presented in Supplementary Figure S1. (**C**) Western blot analysis of protein particles using anti-PhaC antibody. Purified particles were separated by SDS-PAGE (Lane M, SeeBlue Plus2 Pre-Stained Standard; lane 1, control protein particles; lane 2, MAP fusion protein particles) and the presence of antigens was confirmed by Western blot. Full length gel and western blot images are presented in Supplementary Figure S2. (**D**) Solubility analysis of the protein particles. Whole cell lysates from *E. coli* cells expressing protein particles were treated with or without 8 M urea and analysed with SDS-PAGE. Control protein particles (lane 1, untreated whole cell lysate; lane 2, supernatant after centrifugation untreated whole cell lysate; lane 3, whole cell lysate treated with 8 M urea) and MAP fusion protein particles (lane 4, untreated whole cell lysate; lane 5, supernatant after centrifugation untreated whole cell lysate; lane 6, whole cell lysate treated with 8 M urea). Lane M, SeeBlue Plus2 Pre-Stained Standard. A full-length gel image is presented in Supplementary Figure S3. (**E**) Electron microscopy analysis of protein particles. Transmission electron microscope (TEM) image of *E. coli* cells producing protein particles (scale bar: 1 µm) and scanning electron microscope (SEM) image of purified protein particles (scale bar: 3 µm). Unprocessed electron microscopy images are presented in Supplementary Figure S4. (**F**) Flow cytometry analysis of MAP fusion protein particles. Control or MAP fusion protein particles were probed with rabbit anti-rMAP polyclonal antibody (1000-fold dilution). Bound antibody was detected with FITC-labelled secondary antibody (2000-fold dilution). Fluorescence intensities of control (clear plot) and MAP fusion particles (shaded plot) were compared.

Plasmids for production of control protein particles and protein particles displaying MAP fusion antigen were transformed into *E. coli* BL21 cells (Fig. 1A). The transformants were cultured to produce protein particles displaying control and MAP fusion antigen. Analysis of whole cell lysate from control and MAP fusion-producing cells showed that control and MAP fusion antigen bands were dominant. These protein particles were purified successfully by a single centrifugation step at 8000×*g* for 15 min at 4 °C, indicating an easy and relatively simple process of purification (Fig. 1B and Fig S1). The contents also showed some contaminating host cell proteins. In order to confirm absence of production of PHB, both particles producing *E. coli* cells and purified particles were subjected to GC–MS analysis. The results showed that PHB was not detected in *E. coli* and purified particles, indicating that particles did not have the PHB core (data not shown). The protein sequence of the MAP fusion antigen displayed on protein particles was confirmed by subjecting the dominant protein band corresponding to the theoretical molecular weight of MAP fusion antigen to liquid chromatography electrospray ionization tandem mass spectrometric (LC/ESI–MS/MS) (Table S1). Western blot analysis using anti-PhaC antibody demonstrated that PhaC alone and antigen-PhaC fusion were successfully detected in control and MAP fusion protein particles (Fig. 1C and Fig. S2).

In order to evaluate the solubility of overexpressed control and MAP fusion protein particles, the protein particles producing cells were lysed using lysis buffer alone or lysis buffer containing 8 M urea. Following centrifugation, the samples were analysed by SDS-PAGE (Fig. 1D and Fig. S3). PhaC and MAP fusion bands were visible in the supernatant of particles treated with 8 M urea but were not visible in the absence of urea treatment. A faint band of PhaC was also visible in the supernatant of control particles treated with urea. The results suggested that both control and MAP fusion protein particles were insoluble, with control protein particles having some degree of solubility. The protein particles were also observed by electron microscopy (Fig. 1E and Fig. S4). Transmission electron microscopy (TEM) demonstrated the *E. coli* cells were packed with protein particles. Scanning electron microscopy (SEM) on the purified protein particle showed the majority of protein particles had a small size. In addition, the exposure and accessibility of MAP fusion antigens on the surface of protein particles was confirmed in mobilized and immobilized form using flow cytometry and ELISA, respectively (Fig. 1F and Fig. S5). Taken together, these results indicated that MAP-fusion antigen was displayed on the surface of protein particles at high levels and could be easily accessed by immune cells.

In order to produce recombinant proteins, plasmids containing full-length coding sequence for MAP Ag85A, SOD, Ag85B, 74F, and MAP fusion antigen were transformed into *E. coli* BL21 (DE3) and these transformants were cultured to produce recombinant proteins. These recombinant proteins were purified by affinity chromatography. The dominant bands corresponding to the expected molecular weight of the proteins were observed by SDS-PAGE (Fig S6).

### Vaccination with protein particles induced strong MAP-specific serum IgG1 and IgG2a antibody production

**Figure 2:**
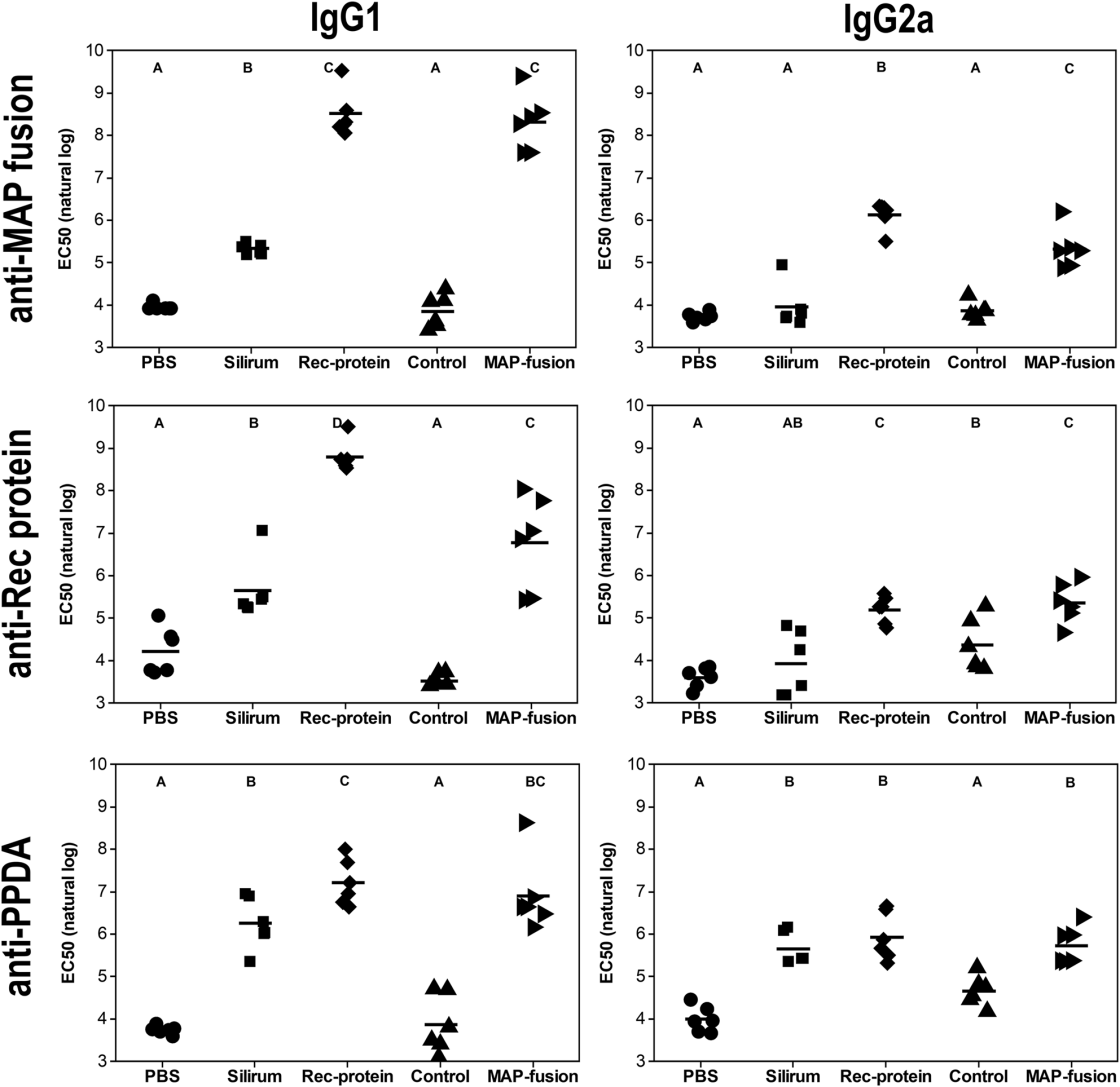
Antibody responses in mice vaccinated with PBS, Silirum, recombinant proteins (Rec-protein), control protein particles (Control), and MAP fusion protein particles (MAP-fusion). Sera were collected three weeks after final vaccination and specific IgG1 isotype and IgG2a isotype antibody responses to recombinant MAP fusion protein; mixture of recombinant proteins (rAg85A, rSOD, rAg85B, and r74F); and PPDA were determined by ELISA and represented as EC50 values. Mean EC50 (natural log) is represented by horizontal lines for each group. Antibody levels were significantly different between the vaccine groups where they do not share a letter (A, B, C or D) with significance cut-off of *P* < 0.05 after using the Benjamini–Hochberg (BH) *P*-value adjustment.

In order to determine whether the MAP fusion protein particle vaccine was capable of inducing antigen-specific antibodies, IgG1 and IgG2a responses against MAP fusion protein, mixture of recombinant proteins, and PPDA were measured in vaccinated mice. PPDA is a crude extract prepared from *M. avium* and contains a wide range of immunogenic proteins of *M. avium*. The mice vaccinated with the MAP fusion protein particles or the recombinant proteins produced significantly higher levels of IgG1 and IgG2a antibodies for MAP fusion protein particle and the recombinant proteins compared to those vaccinated with PBS, control protein particles or Silirum (*P* < 0.05; Fig. 2). Mice vaccinated with MAP fusion protein particles, recombinant proteins or Silirum had significantly higher levels of IgG1 and IgG2a antibodies for the PPDA compared to those vaccinated with PBS or control protein particles (*P* < 0.05). Overall, these results indicated that vaccination with the recombinant proteins or protein particles displaying MAP fusion protein vaccine could induce strong humoral immune responses in mice.

### The protein particle vaccine induced antigen-specific Th-1 responses

**Figure 3:**
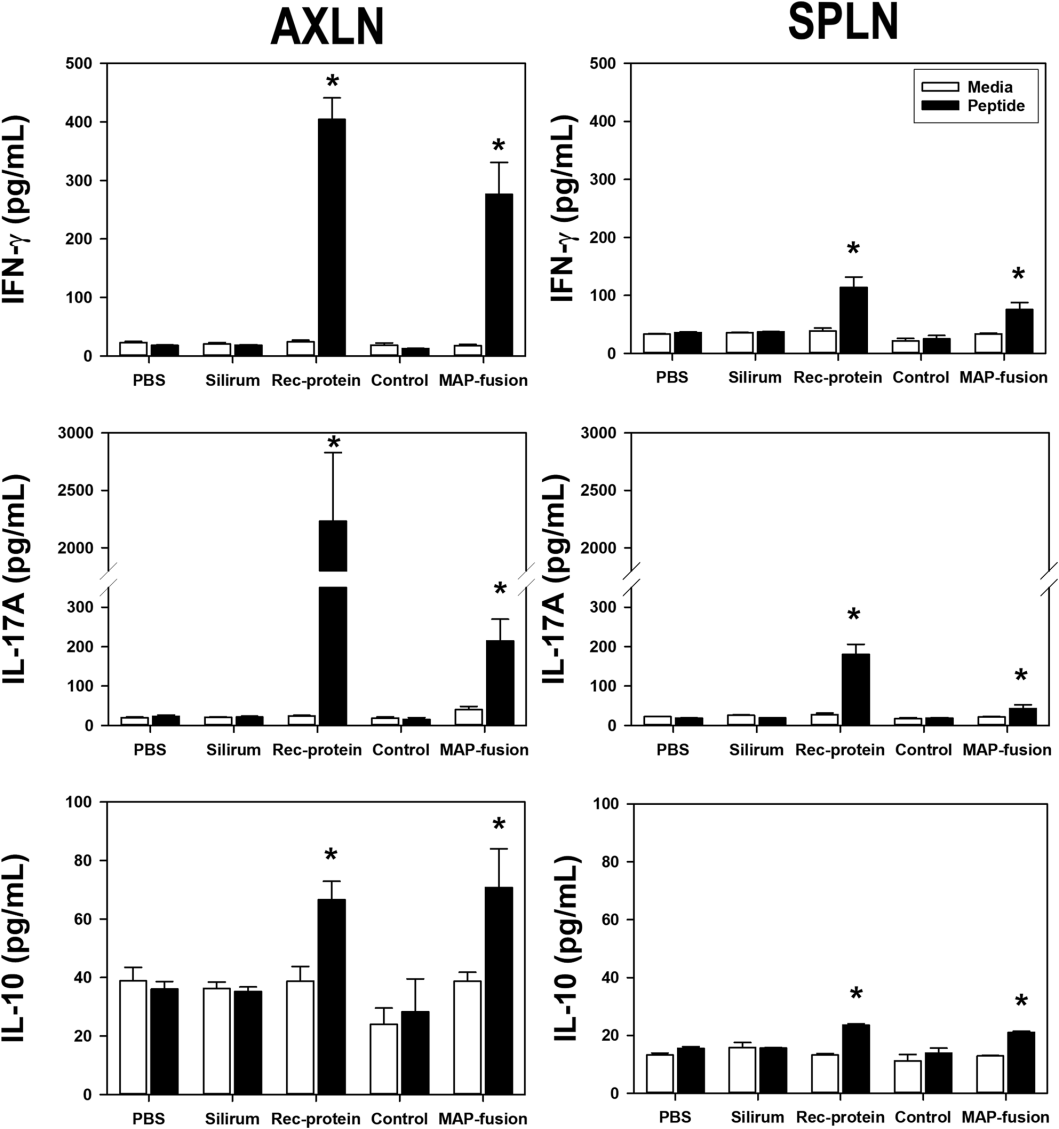
Antigen-specific cytokine responses in mice vaccinated with PBS, Silirum, recombinant proteins (Rec-protein), control protein particles (Control), and MAP fusion protein particles (MAP-fusion). Cells were isolated from AXLN and SPLN of the vaccinated mice and stimulated in vitro with media alone or a mixture of 33 peptides covering Ag85A, SOD, Ag85B, and 74F at a final concentration of 10 µg/mL for 72 h. Cell culture supernatants from AXLN and SPLN were analysed for IFN-γ; IL-17A; and IL-10 production by ELISA. *Significantly different to the PBS group (*P* < 0.05).

In order to determine the potential of MAP-fusion protein particles to induce cell-mediated immunity, immune responses were assessed by measuring production of key cytokines in cells from axillary lymph nodes (AXLN) and spleen (SPLN) from the vaccinated mice. Three weeks post the last vaccination, AXLN and SPLN cells were isolated from the vaccinated mice and re-stimulated in vitro with a pool of Ag85A, SOD, Ag85B, and 74F peptides (Table S2). Mice vaccinated with MAP fusion protein particles or recombinant proteins produced significantly higher levels of IFN-γ, IL-17A, and IL-10 compared with the group of mice vaccinated with PBS (Fig. 3). In addition, both these groups of mice induced higher antigen-specific responses compared with the Silirum-vaccinated group. Mice vaccinated with control protein particles displaying PhaC alone produced minimal cytokine responses which were not different to those in the PBS group (Fig. 3). Based on these results, the control protein particle group was omitted from the main MAP-challenge trial and the PBS group was used as the negative control group. Overall, these results indicated that both the protein particle vaccine displaying MAP fusion antigens and the recombinant protein vaccine induced Th1 cell-mediated immunity in mice.

### mRNA expression analysis in AXLN and SPLN

NanoString technology^21,22^ was used to measure gene expression in immune cells. Initially, 561 immune genes were screened in a subset of mice (n = 3) vaccinated with MAP fusion protein particles and recombinant proteins by investigating mRNA expression in AXLN cells stimulated in vitro with media alone and peptide mixture (data not shown). Twenty-one genes, representing cell-mediated immune responses that demonstrated the strongest responses, were selected for further analyses. In order to determine the involvement of various other immune pathways, mRNA expression of 21 immune effector genes was analysed using Nanostring nCounter technology in AXLN and SPLN stimulated in vitro with a peptide mixture.

**Figure 4:**
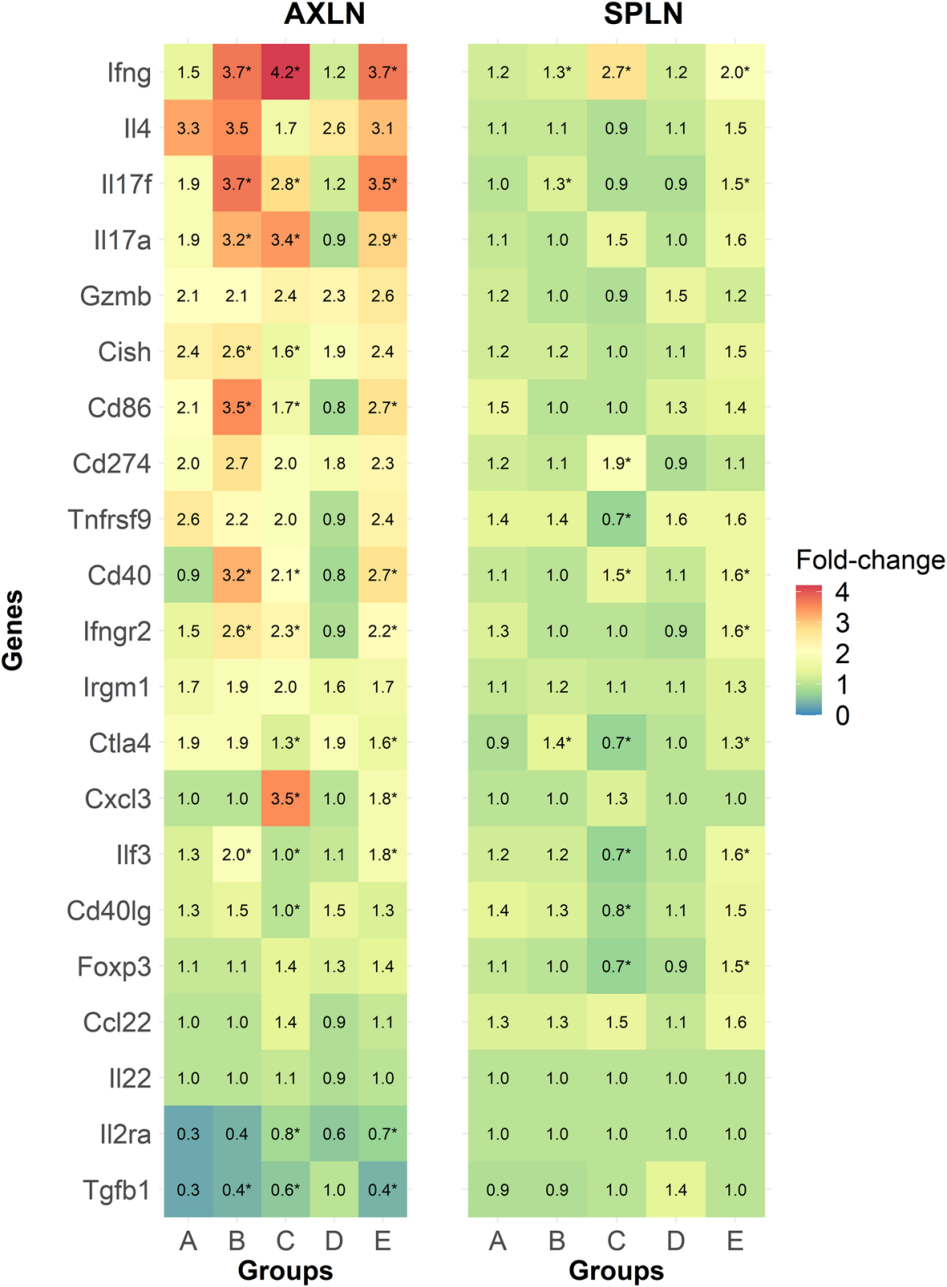
Expression of immune responsive genes in mice vaccinated with PBS (**A**), Silirum (**B**), recombinant proteins (**C**), control protein particles (**D**) and MAP fusion protein particles (**E**). Cells isolated from AXLN and SPLN were stimulated in vitro with media alone or a mixture of 33 peptides covering Ag85A, SOD, Ag85B, and 74F at a final concentration of 10 µg/mL for 72 h. Fold-change was calculated for each gene (peptide mixture/media alone) and data was log transformed for statistical analysis. *Statistical significance compared with the PBS control group (*P* < 0.05).

The expression of immune response genes was differentially modulated in the different vaccine groups (Fig. 4). For example, expression of CD40 was significantly increased in both AXLN and SPLN from mice vaccinated with MAP fusion protein particle, recombinant protein, and Silirum vaccines compared with PBS, suggesting activation of antigen presenting cells (APCs) in these locations (Fig. 4). In addition, CD86 was significantly upregulated in AXLN in the MAP fusion protein particle, recombinant protein, and Silirum-vaccinated mice compared with the PBS control animals, further confirming that MAP fusion protein particle and recombinant protein vaccines were capable of inducing antigen-specific immune responses in mice.

These results also indicated that expression of Th1 cytokines including IFN-γ, IL-17A, and IL17F was upregulated in the MAP fusion protein particle and recombinant protein vaccinated mice compared with the control animals (Fig. 4). These results were in agreement with the results showing significant increases in IFN-γ and IL-17A cytokines in the supernatants of in vitro stimulated cells. Overall, these findings indicated that the MAP fusion protein particle and recombinant protein vaccines can induce Th1 mediated cellular immune responses in mice.

### The protein particle vaccine reduced MAP infection in mice

To assess the ability of MAP fusion protein particle vaccine to elicit protective immunity against MAP infection in mice, the bacterial burden was determined in spleen, liver, and mesenteric lymph node (MLN) tissues of the mice at both 8 and 16 weeks post-challenge. Previously, it has been demonstrated that different vaccines require varying periods of time to clear MAP infection in mice^23^. Thus, two time points post-challenge were chosen to assess vaccine efficacy.

**Figure 5:**
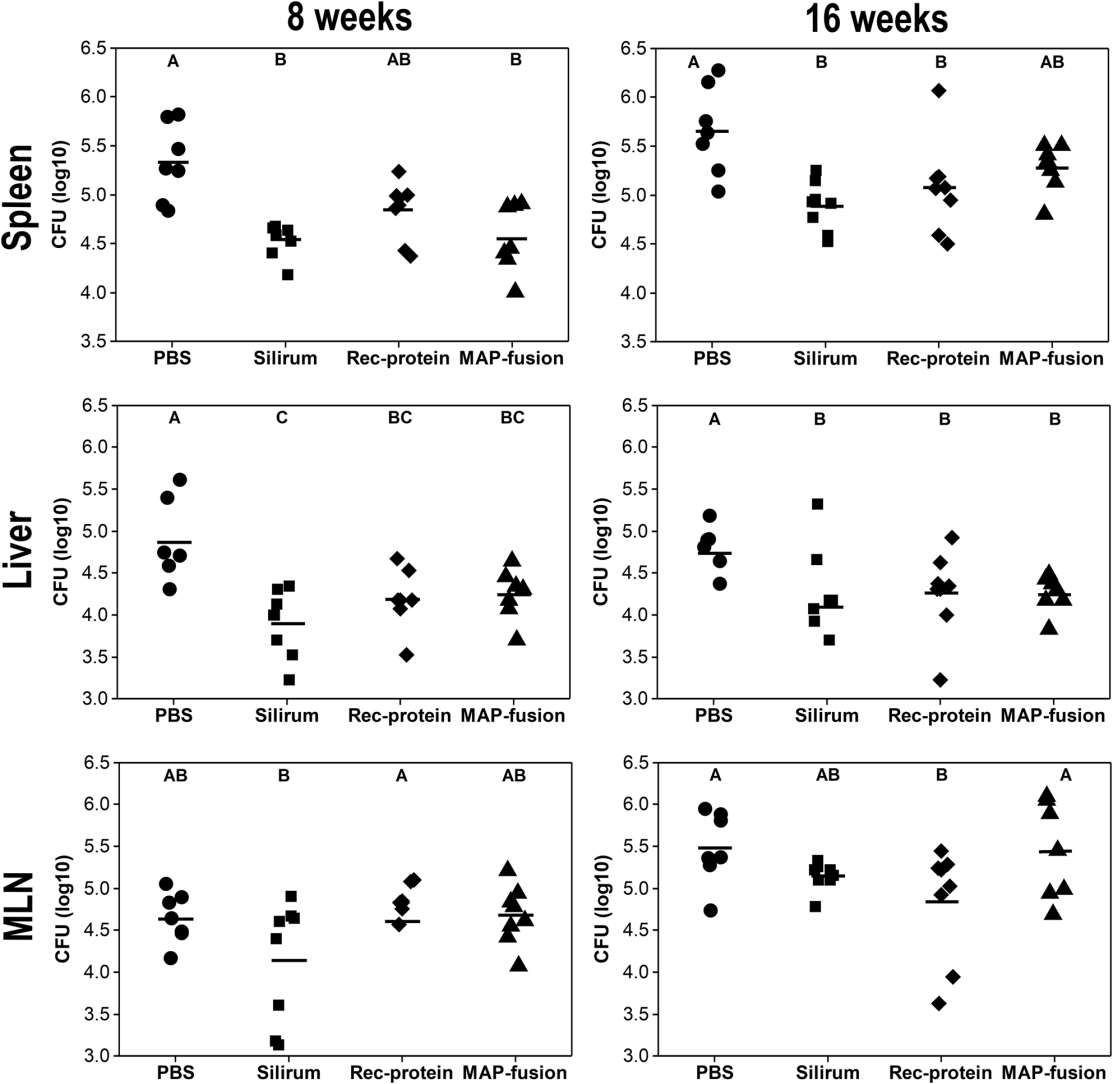
MAP burden following challenge. MAP burden [colony forming unit (CFU log_10_)] was enumerated in spleen, liver, and MLN at 8 weeks and 16 weeks post-challenge. Mean CFU (log_10_) is represented by horizontal lines for each group. MAP burden between the vaccine groups is significantly different where they do not share a letter (A, B, and C) with significance cut-off of *P* < 0.05 after using the BH p-value adjustment.

The results showed that mice vaccinated with the MAP fusion protein particle had significantly lower MAP CFUs in the liver at 8 and 16 weeks post-challenge and in the spleen at 8 weeks compared with those for the PBS group (*P* < 0.05; Fig. 5). Similar significant reductions in MAP burden in the liver and spleen were observed for the recombinant protein and Silirum groups, but in addition, there were significant reductions in the MAP burden in the spleens at 16 weeks for both these groups compared to that for the PBS group (*P* < 0.05). The only significant reduction in the MAP burden in the MLN compared to that for the PBS group was for the recombinant protein group (*P* < 0.05). These results indicated that significant reductions in the MAP burden were observed in selected tissues in mice vaccinated with the MAP fusion protein particles, the recombinant proteins and Silirum vaccine.

**Figure 6:**
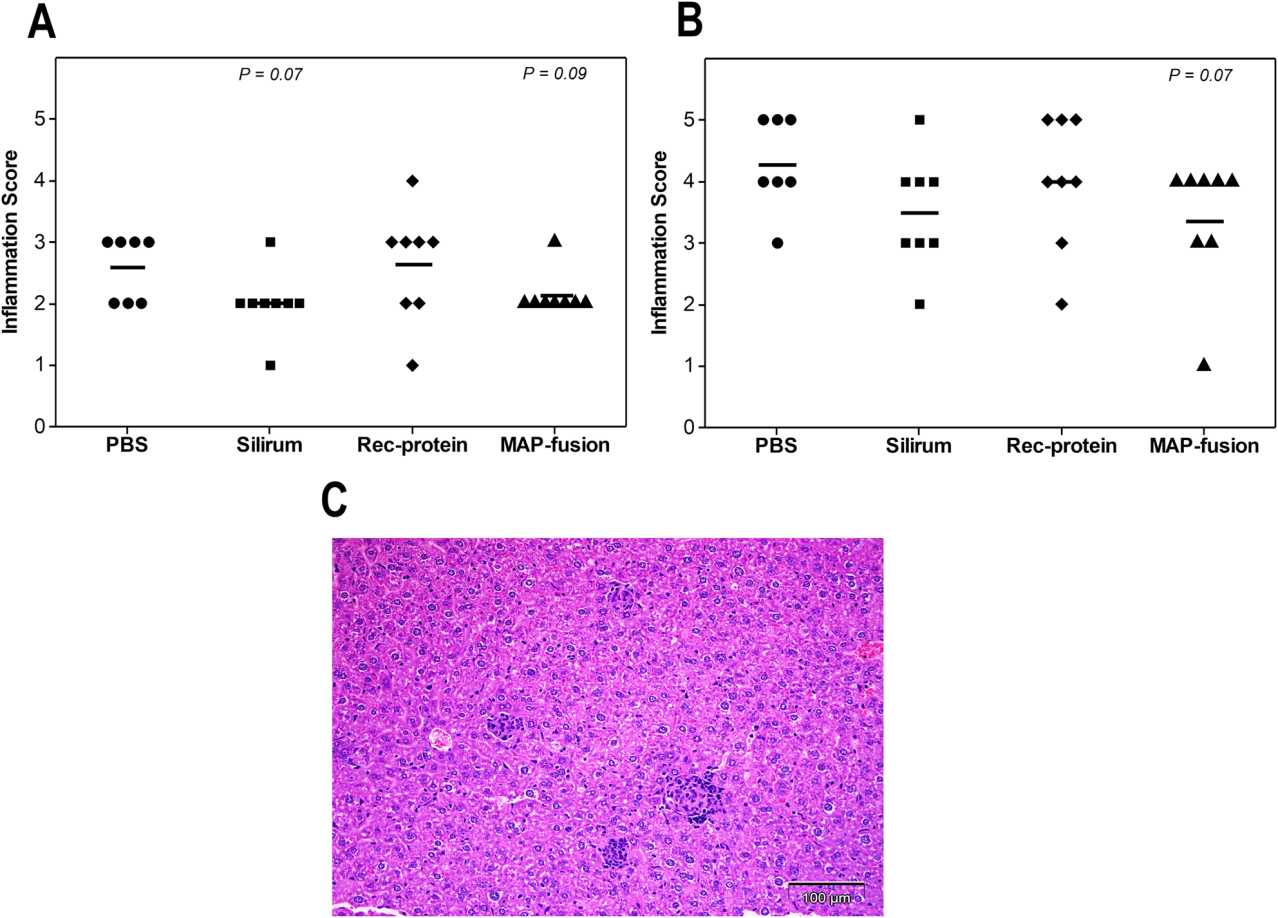
Histopathological examination of the mice post-challenge with MAP. The tissue sections were stained with haematoxylin and eosin and Ziehl–Neelsen stains and examined by light microscopy. Small (up to 25 µm in diameter) and large granuloma (up to 100 µm in diameter) counts were measured in the liver at 8 and 16 weeks post-challenge. (**A**) The inflammatory score ranged from 0 to 5 based on the total granuloma score, with inflammatory score of 1: very low granuloma score to 5: high granuloma score. The total granuloma score was the sum of the scores for the small and large granulomas. Small granuloma (up to 25 µm in diameter) containing predominantly small lymphocytes were recorded as 1 and large granulomas (up to 100 µm in diameter) containing predominantly epithelioid macrophages as 2. Mean inflammation score is represented by horizontal lines. (**B**) A photograph of liver section was obtained from PBS vaccinated mice showing small and large granulomas randomly dispersed in the liver. Bar represents 100 µm.

To assess histopathological changes in the mice post-challenge, liver sections from the mice were examined following staining with haematoxylin and eosin and Ziehl–Neelsen stains. The severity of the liver pathology at 8 and 16 weeks showed a similar trend to the MAP culture results. A trend of reduced inflammation of the liver was observed in MAP fusion protein particle-vaccinated mice compared to the PBS group at 8 and 16 weeks (*P* = 0.09 and *P* = 0.07) post-challenge, although these were not statistically different (Fig. 6A). The challenged animals had randomly dispersed granulomas with central epithelioid macrophages surrounded by lymphocytes (Fig. 6B). Ziehl–Neelsen staining of the liver sections revealed small numbers of acid-fast bacilli within macrophages in the granulomas.

## Discussion

MAP infection causes considerable losses to livestock industries. Commercially available vaccines can limit progression of infection and reduce shedding of the organism, but they can interfere with the on-farm TB surveillance programme^7,24^. In the current study, we demonstrated that a vaccine comprised of protein particles expressing MAP fusion antigens Ag85A, SOD, Ag85B, and 74F induced antigen-specific immune responses in mice, and reduced the severity of MAP infection in selected tissues following challenge with MAP. Significant reductions in the MAP burden were observed in the MAP fusion protein particle group for both the liver and spleen at 8 weeks post-challenge and in the liver tissues at 16 weeks post-challenge. The only vaccinated group to show a significant reduction in the MAP burden in the MLN was the recombinant protein group at 16 weeks post-challenge. The absence of a consistent reduction in the MAP burden in MLN may be due to the high challenge dose (7.5 × 10^7^ CFU) of a virulent strain of MAP, together with a non-natural challenge route, intraperitoneal. In a review of models to assess MAP vaccine efficacy, the limitations of the mouse model were noted and the recommended readout in mice were induction of antigen-specific immune responses and colonization (CFU) of the liver and spleen at 6, 12 and 18 weeks following an intraperitoneal challenge with a virulent strain of MAP^20^. Consistent with this, the findings in the current study showed that MAP fusion protein particle vaccine induced strong cell-mediated and humoral responses and reduced severity of MAP infection as indicated by reduction in MAP bacteria and granulomas in the vaccinated mice. While a previous study has demonstrated that two separate Salmonella vectors expressing Ag85A-SOD-Ag85B and 74F could reduce MAP burdens in mice following challenge^19^, this is the first report to demonstrate efficacy of MAP Ag85A-SOD-Ag85B-74F antigens as a fusion protein displayed on protein particles.

Cell-mediated immune responses are considered most effective in clearing and providing protection against intracellular pathogens such as mycobacteria^25,26^. Th1-associated cytokines produced by CD4^+^ and CD8^+^ T-cells including IFN-γ, TNF-α, and IL-17A have shown to correlate with protection against mycobacterial pathogens^27–29^. In the current study, we observed that mice vaccinated with MAP fusion protein particles or recombinant proteins exhibited antigen-specific IFN-γ responses, which may have played a critical role in providing protective immunity against MAP infection. These findings were in agreement with previous reports and suggest that protection against MAP challenge in mice correlated with antigen-specific IFN-γ immune responses^19,30^. Higher antigen-specific production of the pro-inflammatory cytokine IL-17A was also observed in MAP fusion protein particles or recombinant protein vaccinated mice compared with control mice. IL-17A is mainly produced by T helper 17 cells (T_h_17) and contributes to clearance of pathogens from mucosal surface^31^. It has been demonstrated that γδ T-cells also participate in host immune defence by producing IL-17A in response to mycobacterial infection^27,28,32^. Although, there are suggestions that humoral immunity may be important against *Mycobacterium tuberculosis*^33^, the role of humoral immunity against MAP infection in ruminants has not been confirmed. However, in the current study, strong antibody responses to recombinant protein, MAP fusion protein, and PPDA were detected in mice vaccinated with MAP fusion protein particles or recombinant proteins. Overall, these findings indicated that MAP fusion antigens induced both antigen-specific cell-mediated and humoral responses.

Particle vaccines have several attractive features and have been reported to induce better immune responses compared to soluble antigens by primarily targeting APCs and activating CD4^+^ and CD8^+^ T cells. While the different cell types were not characterised in the current study, the high expression of CD40 and CD86 in ALXN and SPLN from mice vaccinated with MAP fusion protein particles suggested that the MAP proteins were taken up by the APCs. CD40 and CD86 are costimulatory proteins present on APCs and play a key role in activating T-cell mediated immune responses by presenting processed antigens to CD4^+^ T cells. Previous studies have demonstrated the role of CD40 and CD86 co-stimulatory proteins in immune cells in MAP infection^34,35^.

Several additional mechanisms have been proposed for how particulate antigens induce stronger immune responses than soluble antigens^36,37^. Particle size in the range of 0.05–0.5 µm is considered optimal for effective uptake by APCs and eliciting potent antigen-specific immune responses^38,39^, allowing prolonged cross-presentation of antigens by both MHC I and II to CD4^+^ and CD8^+^ T cells. The resulting immune responses may be preferable to those induced by soluble antigens, which are predominantly cross-presented to MHC class II in endosomes. The observed size of the majority of the MAP fusion protein particles was 0.5 µm or less and therefore, the particles are likely to be taken up readily by professional APCs. In the current study, this was evident by an increased expression of co-stimulatory proteins CD40 and CD86 and enhanced production of IFN-γ and IL-17A from both AXLN and SPLN. Autophagy is another possible mechanism which could play a crucial role against intracellular pathogens^40,41^. In autophagy, proteins are delivered to lysosomes for degradation and antigen processing, followed by presentation in DCs, which has been shown to improve vaccine efficiency. BCG-vaccine containing Ag85B immunogenic peptide has been demonstrated to enhance antigen-specific MHC class II presentation by DCs via autophagy, which resulted in increased vaccine efficacy and thus providing better protection against *M. tuberculosis* compared to a non-autophagy activating vaccine^42,43^. The MAP fusion protein particle vaccine developed here also contained Ag85A, Ag85B and Ag85C, which possibly resulted in induction of autophagy in these vaccinated mice. Alternatively, it is possible that APCs effectively presented the MAP fusion antigen to T cells such as γδ and T_h_17 cells, resulting in production of antigen-specific IFN-γ and IL-17^27,32^. Further studies will be required to determine which T cells are induced by MAP fusion protein particles.

Interestingly, Silirum-vaccinated mice only showed an increase in mRNA levels of IFN-γ and IL-17A but no increase in protein, yet these mice had a reduced MAP infection in various tissues. It has been reported that mRNA levels can correlate poorly to protein levels and efficient protein translation depends on various factors such as translation rates, post-translational processing and cell cycle state^44,45^. It is possible that only small amount of RNA was converted into IFN-γ and IL-17A protein levels in mice vaccinated with Silirum, which was below the detection limit of the assay. Additionally, the Silirum vaccine contains only low amounts of Ag85A, SOD, Ag85B, and 74F, which resulted in lower peptide mixture-specific IFN-γ and IL-17A responses compared with responses in the MAP fusion particle and recombinant protein vaccinated mice. Stimulation of cells from the Silirum-vaccinated mice with PPDA would have provided better understanding of mycobacterial antigen-specific cellular immune response, however results were not available for this antigen.

Various studies have demonstrated that fusion proteins are a better strategy compared to individual recombinant proteins to develop effective vaccines^46,47^. For example, fusion proteins eliminate the limitation of epitope restriction as multiple immunogenic domains from several proteins can be combined in a single molecule. Fusion strategy also allows increased production and secretion of smaller domains of antigens compared to full-length proteins^47,48^. Several MAP specific antigens have been used as fusion proteins subunit-based vaccines and in a number of studies a reduction of MAP infection in mice has been demonstrated^30,49^. In the current study, we produced protein particles expressing MAP specific domains of Ag85A, SOD, Ag85B, and 74F antigens as a fusion protein and evaluated their ability to induce protection in mice against MAP challenge. These antigens have been shown to participate in various cellular processes in MAP bacteria. For example, the Ag85 family of proteins are involved in mycobacterial cell wall biogenesis and bind to fibronectin protein and have been demonstrated to induce strong T-cell immune responses^50^. SOD has been shown to protect against oxidative stress during infection as well as tissue damage and can elicit strong cellular immune responses^51,52^. The 74F protein is a fusion of Map3527, a trypsin-like serine protease and Map1519, a hypothetical protein^30^. Previous reports have shown that 74F antigen can induce Th1 and Th2 immune responses (IFN-γ and IL-17A) and provide protective immunity when used as a recombinant protein as well as expressed as part of the secretory system of a Salmonella vector in combination with MAP Ag85A, Ag85B, and SOD antigens^19,30^. However, MAP antigens expressed as a fusion of Ag85A, Ag85B, and SOD without the addition of 74F failed to provide complete protection against MAP infection^53^. Our results also demonstrated that both a mixture of four soluble antigens as recombinant proteins or protein particles expressing fusion of Ag85A, SOD, Ag85B, and 74F resulted in a similar level of protection against MAP challenge. Based on these observations, it is conceivable that truncated 74F in the antigen mixture could be essential for inducing immunity against MAP infection.

For production of protein particulate vaccines, *E. coli* can be propagated easily in the laboratory and vaccine production is scalable to produce large quantities of protein particles. Bioengineered *E. coli* accumulated MAP fusion protein particles up to 9.6% (wt/wt) of their total biomass wet weight (data not shown). This high level of expression in *E. coli*, together with simple purification procedures, consisting of only lysis and centrifugation steps, suggest that protein particles can be produced cost effectively and provide an attractive platform to display antigens with the ability to induce potent immune responses. Although particulate vaccines have attractive attributes relating to ease of production and cost^36,54^, some nanomaterials derived particulate vaccines have a toxicity concern. This can be because of problems with their biocompatibility, biodegradable nature and their transfer across the placental and blood–brain barrier^55^, making them unsuitable for animal and human use. In contrast, particulate vaccines derived of proteins, virus-like particle, and polymeric particles are of biological origin and composed entirely of biodegradable materials (proteins, poly-lactic acid and chitosan) which are biocompatible and are non-toxic. In the current study there was minimal inflammatory response at the vaccination site for the MAP fusion protein particle vaccine, indicating low toxicity of this vaccine.

In summary, this report provides evidence that MAP antigens Ag85A^202–347^-SOD^1–72^-Ag85B^173–330^-74F^1–148+669–786^ expressed as a fusion on protein particles can induce strong cellular and humoral responses and can reduce the bacterial burden following MAP challenge in mice. The defined nature of the antigens in the protein particle vaccine makes it unlikely that the vaccine will interfere with the current bovine tuberculosis skin test when used in cattle. However, further studies in target species such as cattle will be required to evaluate whether the protein particles-based vaccine can confer protective immunity against Johne’s disease without inducing responses to the bovine tuberculosis skin test.

## Materials and methods

### Animals

Six to eight weeks old female BALB/c mice were used in the study and were kept in a biosecurity containment-2 room at the Ulyatt Reid Small Animal Facility, Grasslands, AgResearch. All animal experiments were performed in compliance with the Animal Welfare Act 1999 (the Act) and the Animal Welfare (Records and Statistics) Regulations 1999 and approved by the Grasslands Animal Ethics Committee, AgResearch.

### Bacterial strain

A virulent strain of MAP (W09/710) previously isolated from a JD infected cow ^56^ was used to challenge the mice. MAP was grown to mid-log phase in Middlebrook 7H9 broth (Difco, Detroit, MI) supplemented with 10% Oleic acid albumin dextrose complex (OADC), 0.5% glycerol (Sigma, St. Louis, MO), 0.05% Tween 20 (Sigma, St. Louis, MO), and 2 µg/mL mycobactin J (Allied Monitor, Inc., Fayette, MO) for 8 weeks. The MAP culture at passage 9 was harvested by centrifugation at 4000×*g* for 15 min and washed twice with PBS (10 mM, pH-7.4). The organisms were then diluted in 10 mL of PBS and de-clumped using a high-performance ultrasonic cleaning system. The concentration of the bacteria was adjusted to the required challenge dose.

### Generation of plasmid construct to produce protein particles displaying MAP fusion antigen and purification of protein particles

All plasmids and oligonucleotides used in the current study are described in Table 1. The pPolyN plasmid system^48^ was used to ligate the MAP fusion antigen to the N-terminal of PhaC protein. The coding sequence for the MAP fusion antigen, including restriction endonuclease sites for cloning, was amplified from the pET151 MAP fusion plasmid with codon-optimised MAP fusion antigen-NdeI-F and MAP fusion antigen-SpeI-R primers using Expand Hi fidelity PCR system (Sigma, St. Louis, MO). The amplified PCR product was cloned into a pCR-II Zero Blunt TOPO vector (ThermoFisher Scientific, New Zealand). The MAP fusion antigen sequence was excised from the resultant plasmid using NdeI and SpeI restriction enzymes (Sigma, St. Louis, MO) and was ligated into pPolyN plasmid digested with the same enzymes. The plasmid, containing MAP fusion antigens fused to N-terminal of PhaC protein (pPoly-MAP fusion) was transformed into *E. coli* BL21 (DE3) cells (ThermoFisher Scientific, New Zealand). Empty pPolyN plasmid was also transformed into *E. coli* BL21 (DE3) cells to produce control (PhaC alone) protein particles.

**Table 1:** Plasmids and oligonucleotides used in the present study.

Plasmid and oligonucleotides	Description	Source
**Plasmids**		
pPoly-N-PhaC	pET-14b derivative containing phaC from *Ralstonia eutropha* and SpeI site upstream of phaC to clone antigen of interest, Amp^r^	^48^
pPoly-MAP-fusion-PhaC	pPoly-N-PhaC derivative containing MAP Ag85A^202–347^-SOD^1–72^-Ag85B^173–330^-74F^1–148+669–786^ MAP-fusion antigen inserted into NdeI/SpeI sites,	This study
pET151-6His-MAP-fusion	pET-151 containing MAP Ag85A^202–347^-SOD^1–72^-Ag85B^173–330^-74F^1–148+669–786^ as MAP-fusion antigen, Amp^r^	GeneArt
pET151-6His-Ag85A	pET-151 containing full length MAP Ag85A with a 6xHis tag upstream, Amp^r^	GeneArt
pET151-6His-Ag85B	pET-151 containing full length MAP Ag85B with a 6xHis tag upstream, Amp^r^	GeneArt
pET151-6His-SOD	pET-151 containing full length MAP SOD with a 6xHis tag upstream, Amp^r^	GeneArt
pET151-6His-74F	pET-151 containing full length MAP 74F with a 6xHis tag upstream, Amp^r^	GeneArt
**Oligonucleotides (5′–3′)**		
MAP-fusion-NdeI-F	TATATACATATGGGTCCGAGCCTG	This study
MAP-fusion-SpeI-R	ATATATACTAGTCCTGCAACTGC	This study

Protein particles displaying MAP fusion antigen fused to PhaC and control (PhaC alone) protein particles were produced in *E. coli*. Cultures of genetically modified *E. coli* BL21 (DE3) were grown in LB supplemented with 75 µg/mL ampicillin in a shaking incubator at 37 °C. The cultures were induced with 0.5 mM of isopropyl β-D-1-thiogalactopyranoside (IPTG) (Sigma, St. Louis, MO) until an OD_600_ of 0.5 was reached, after which, the cultures were further incubated for 48 h at 25 °C with shaking at 200 rpm.

*E. coli* cells producing protein particles were pelleted by centrifugation at 5000×*g* for 15 min at 4 °C and re-suspended in PBS (10 mM, pH-7.4). The cells were disrupted with a microfluidizer and the lysate was centrifuged at 8000×*g* for 15 min at 4 °C to harvest the protein particles. These were washed twice with cold PBS and then washed with PBS + 2 M urea followed by PBS + 1% (v/v) Triton X-100. Although mechanical disruption using the microfluidizer is very efficient in lysing all bacteria, the protein particles were treated with 70% ethanol for 1 h in order to kill any residual bacteria. The protein particles were again washed twice in cold PBS and re-suspended in PBS as a 20% slurry. The resulting protein particles were plated on LB medium to confirm sterility of the suspension.

### Flow cytometry

A 50 µg portion of purified control and MAP fusion protein particles was washed twice with flow cytometry buffer (PBS containing 1% fetal bovine serum). The protein particles were incubated with 1000-fold diluted polyclonal rabbit anti-rMAP antibodies for 30 min at room temperature with mixing. After the incubation, the protein particles were washed in flow cytometry buffer and stained with fluorescein isothiocyanate labelled goat anti-rabbit IgG antibody (MP Biomedicals, CA) for 30 min at 4 °C in the dark. After washing, the protein particles were run on a BD FACSVerse flow cytometer to collect minimum of 100,000 events for each sample. The data was analysed using FlowJo software 10.2.

### Production and purification of recombinant proteins

Full-length genes encoding for MAP Ag85A, SOD, Ag85B, 74F, and MAP fusion antigens, each with a 6 × histidine tag were synthesised by GeneArt (ThermoFisher Scientific, Auckland) and cloned into a pET151 plasmid expression vector. The resultant plasmid constructs were transformed into *E. coli* BL21 (DE3) cells and the transformants were selected on LB media supplemented with 100 µg/mL ampicillin (Sigma, St. Louis, MO). Recombinant Ag85A, SOD, Ag85B, 74F, and MAP fusion antigen proteins were produced as his-tagged proteins by inducing the cultures with 0.5 mM IPTG for 4 h. After the incubation, cells were pelleted by centrifugation at 6000×*g* for 15 min at 4 °C. Cells were re-suspended in PBS buffer and disrupted using a microfluidizer (Microfluidics M-110P, Westwood, MA). Inclusion bodies were recovered by centrifugation at 15,000×*g* for 15 min at 4 °C. His-tagged proteins were purified by affinity chromatography using gravity flow nickel-chelate (Ni-NTA) columns following the manufacturer’s instructions (Takara Bio, CA, USA). Eluted fractions were analysed by SDS-PAGE and the fractions containing the desired proteins were pooled and dialysed against PBS.

Triton X-114 (Sigma, St. Louis, MO) has been shown to deplete endotoxin contamination from recombinant protein preparations^57^. Each of the dialysed recombinant proteins were treated with Triton X-114 in order to further reduce endotoxin contamination as described previously^57^. Briefly, Triton X-114 was added to the protein solution at a final concentration of 1% (v/v). This suspension was continuously mixed at 4 °C for 30 min to obtain a homogenous suspension followed by further 10 min incubation at 37 °C. The mixture was centrifuged at 20,000×*g* for 10 min at room temperature to separate the phases. The upper phase containing the protein was transferred to a fresh tube and the extraction procedure was repeated three more times.

### Vaccination of mice

The first four vaccines, namely (1) a mixture of MAP recombinant Ag85A, SOD, Ag85B, and 74F proteins (12.5 µg of each protein); (2) control protein particles (50 µg); (3) MAP fusion protein particles (50 µg); and (4) PBS; were formulated in Emulsigen-D (20%, vol/vol, MVP Laboratories, Omaha, NE). A fifth vaccine was Silirum vaccine containing heat-inactivated MAP strain 316F (Zoetis, NSW, Australia). A total of 110 BALB/c mice were divided into five groups, each group containing 22 animals (six for immunological studies and 16 for challenge studies). The animals receiving the four vaccines formulated with Emulsigen-D were vaccinated three times by the subcutaneous route at the back of the neck with a 200 µL dose of vaccine at a 2-week interval. The mice vaccinated with Silirum vaccine were vaccinated subcutaneously at the site, but only received a single dose contained in a 100 µL volume.

### In vitro re-stimulation assay and cytokine ELISA

Three weeks after the last vaccination, six mice from each group were euthanized. Mice were deeply anaesthetized using a subcutaneous injection of 300 µL of ketamine/medetomidine 2.53 mg/mL Ketamine (Ketamine Injection; Parnell Laboratories NZ Ltd, Auckland, New Zealand; Product No. RVM A005925) and 0.03 mg/mL Medetomidine hydrochloride (Domitor; Zoetis NZ, Auckland, New Zealand; Product No: RVM A06177) to collect blood by cardiac heart puncture. Immediately afterwards mice were euthanized by use of CO_2_ inhalation and cervical dislocation. Euthanized mice were dissected, and spleens were removed to prepare a single cell suspension from spleen (SPLN) as described previously^48^. In addition, a single cell suspension was also prepared from axillary lymph nodes (AXLN). The AXLN were removed and ground with a plastic pestle in a 1.5 mL tube in volume of 0.5 mL DMEM supplemented with 10% heat inactivated fetal bovine serum, penicillin–streptomycin and sodium pyruvate (ThermoFisher Scientific, New Zealand). The suspension was passed through a 70 µm sterile cell strainer to remove tissue debris and washed with DMEM supplemented with 10% heat inactivated fetal bovine serum, penicillin–streptomycin, and sodium pyruvate (Complete DMEM, cDMEM).

SPLN and AXLN from each mouse were re-suspended in cDMEM and plated in U-bottom 96-well plates at final densities of 0.25 × 10^6^ and 1 × 10^6^ cells per well, respectively. The cells were stimulated for 3 days with either medium alone or with a mixture of 33 peptides covering Ag85A, SOD, Ag85B, and 74F antigens (Table S2) pooled together and used at a final concentration of 10 µg/mL (GenScript, NJ, USA) in a final volume of 200 µL at 37 °C with 5% CO_2_. Following incubation, culture supernatants were carefully recovered by centrifugation at 250×*g* for 10 min and stored at − 20 °C until analysed for cytokine release by ELISA. Cell lysis buffer (RLT, Qiagen) was added to the cell pellet and the plates were stored at − 80 °C until RNA isolation was undertaken.

Levels of antigen-specific IFN-γ and IL-10 were measured in culture supernatants by sandwich ELISA according to the manufacturer’s instructions (BD Biosciences, San Diego, CA). Antigen-specific IL-17A concentrations were measured by sandwich ELISA kit following the manufacturer’s instructions (BioLegend, San Diego, CA).

### Measurement of antigen-specific antibodies in serum

Sera were collected from mice 3 weeks after final immunization to measure MAP fusion, recombinant protein and *M. avium* protein purified derivatives (PPDA)-specific immunoglobulin (IgG), IgG1 and IgG2a antibodies by ELISA. Briefly, 250 ng/well of MAP fusion recombinant protein, mixture of four recombinant proteins (62.5 ng each), or PPDA (Prionics, Schlieren, Switzerland) were used for coating Microlon high-binding plates (Greiner Bio-One, Germany). Sera from individual mice were diluted and added to the plates and incubated for 2 h at room temperature with shaking at 100 rpm. After the incubation, the plates were washed four times with washing buffer (0.5% Tween-20 in PBS) with 3 min soaking during each wash. Diluted goat anti-mouse IgG1 or IgG2a antibodies conjugated with horseradish peroxidase (ICLLab, USA) were added to the plates and incubated for 1 h at room temperature with shaking. The plates were washed and 3, 3′, 5, 5′-Tetramethylbenzidine (TMB) substrate (BD Biosciences, San Diego, CA) was added to each well and incubated for 30 min at room temperature. The reaction was stopped by adding 0.5 M H_2_SO_4_ to well and the OD at 450 nm was measured using a microplate reader (VERSAmax; Molecular Devices, Sunnyvale, CA, USA) to detect the IgG isotypes. Data are presented as half maximal effective concentration (EC50) values in response to MAP fusion antigen recombinant proteins (Ag85A, SOD, Ag85B, and 74F) or PPDA.

### Nanostring analysis of mRNA measurement

Total RNA was isolated from in vitro stimulated AXLN and SPLN cells using an RNeasy kit following the manufacturer’s instructions (Qiagen, Germany). RNA samples were analysed using an nCounter Mouse Immunology Panel and PlexSet-24 on the nCounter Analysis System as described previously^58^.

### Challenge of mice and MAP culture for counting colony forming unit (CFU)

Three weeks after the final vaccination, the remaining mice in the PBS, Silirum, recombinant protein and MAP fusion protein particle groups were challenged intraperitoneally with 7.5 × 10^7^ CFU of MAP bacteria in a 0.2 mL volume, a dose optimized for mice in a preliminary study (data not shown). Eight mice from each group were euthanized by cervical dislocation at 8 weeks post-challenge and the remaining eight animals at 16 weeks post-challenge. Spleens, livers, and mesenteric lymph nodes (MLN) were removed aseptically to enumerate bacterial load in these tissues. To do this, spleen, liver, and MLN tissues were individually homogenized in 3 mL of PBS + 0.5% Tween 80 using a Seward Stomacher 80 (Seward, Norfolk, UK). These homogenates were plated in tenfold dilutions on Middlebrook 7H11 agar supplemented with 10% OADC, glycerol (0.5%), Tween 20 (0.05%), and mycobactin J (2 µg/mL). The plates were sealed with Sellotape and incubated at 37 °C for 8 weeks before counting the colonies.

### Histopathology

Sections of liver were fixed in 10% buffered formalin, embedded in paraffin wax, sectioned at 4 µm, stained with haematoxylin and eosin and Ziehl–Neelsen stains and examined by light microscopy. The inflammatory score was initially determined by producing a granuloma score which consisted of counting the number of granulomas in the liver section with pathologist blinded to the vaccination groups. The inflammatory score ranged from 0 to 5 based on the total granuloma score, with inflammatory score of 0: no granuloma score, 1: very low granuloma score, and 5: high granuloma score. The total granuloma score was the sum of the scores for the small and large granulomas. A small granuloma (up to 25 µm in diameter and containing predominantly small lymphocytes) was received a score of 1, while large granulomas (up to 100 µm in diameter) containing predominantly epithelioid macrophages received a score of 2. The presence of acid-fast bacteria is the sections was assessed by Ziehl–Neelsen staining.

### Statistical analysis

Statistical significance of the differences between the treatment groups was determined by two-way analysis of variance (ANOVA) using R statistical software package and interaction with multiple comparisons including cytokines and MAP CFU was performed on log scale with *P*-value adjusted by the Benjamini–Hochberg method^59^. Similarly, EC50 data for IgG1 and IgG2a was transformed to natural log for statistical analysis data. A *P* value of < 0.05 was considered significant.

## Supplementary Information

Supplementary Information.
