# Supplementary material for: Self-assembled particulate vaccine elicits strong immune responses and reduces Mycobacterium avium subsp. paratuberculosis infection in mice
Source: Sci Rep. 2020 Dec 18;10:22289. doi: 10.1038/s41598-020-79407-7 (PMC7749150; doi:10.1038/s41598-020-79407-7)
Supplement: Supplementary file 1 — Supplementary Information. [file 41598_2020_79407_MOESM1_ESM.docx]

**Self-assembled particulate vaccine elicits strong immune responses and reduces *Mycobacterium avium* subsp. *paratuberculosis* infection in mice**

Sandeep K Gupta^1*^, Natalie A Parlane^1^, Dongwen Luo^2^, Bernd H A Rehm^3, 4^, Axel Heiser^1^, Bryce M Buddle^1^ and D Neil Wedlock^1^

^1^AgResearch, Hopkirk Research Institute, Palmerston North, New Zealand.

^2^Bioinformatics and Statistics, AgResearch, Palmerston North, New Zealand.

^3^Centre for Cell Factories and Biopolymers, Griffith Institute for Drug Discovery, Griffith University, Brisbane, QLD, Australia.

^4^Menzies Health Institute Queensland (MHIQ), Griffith University (Gold Coast Campus), Australia.

***Corresponding Author:**

Sandeep K Gupta

AgResearch, Hopkirk Research Institute, New Zealand

Grasslands Research Centre,

Private Bag 11008, Palmerston North 4442,

Tel: +64 6 353 8697

Fax: +64 6 353 7853

Email: [sandeep.gupta@agresearch.co.nz](mailto:sandeep.gupta@agresearch.co.nz)

**Supplementary information**


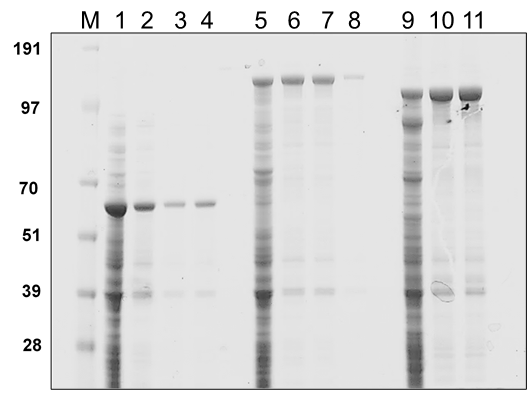


**Figure S1.** Uncropped version of the image used in Figure 1B. SDS-PAGE analysis of purified protein particles produced in *E. coli*. Lane M, SeeBlue Plus2 Pre-Stained Standard; lane 1, whole cell lysate from control protein particles produced in *E. coli*; lane 2, purified control protein particles (10 µL); lane 3, purified control protein particles (5 µL); lane 4, purified control protein particles (2.5 µL); lane 5-8, 74F full length protein particles (not used in the current study); lane 9, whole cell lysate from MAP fusion protein particles produced in *E. coli*; lane 10, purified MAP fusion protein particles (10 µL); lane 11, purified MAP fusion protein particles (5 µL).


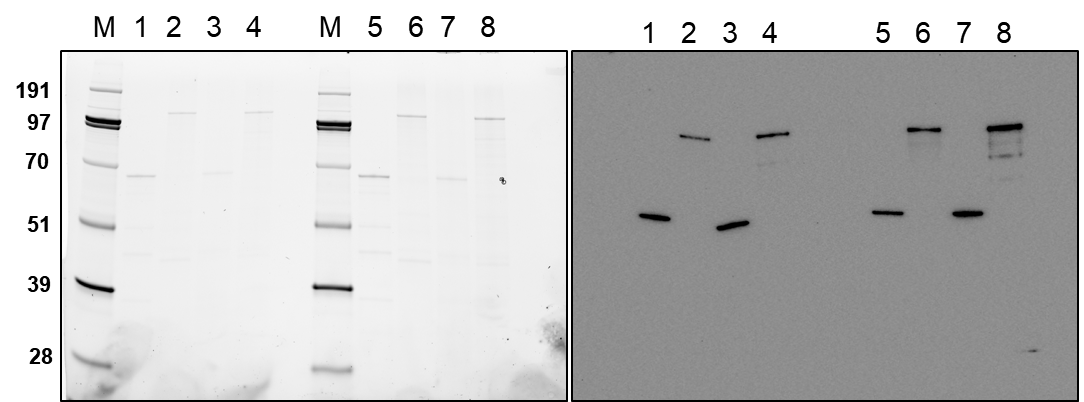


**Figure S2.** Uncropped version of the image used in Figure 1C. Western blot analysis of protein particles using anti-PhaC antibody. Purified particles were separated by SDS-PAGE (Lane M, SeeBlue Plus2 Pre- Stained Standard; lane 1, control protein particles (1 µL); lane 2, MAP fusion protein particles (1 µL); control protein particles (2 µL); lane 2, MAP fusion protein particles (2 µL). The presence of antigens was confirmed by Western blot using anti-PhaC antibodies.


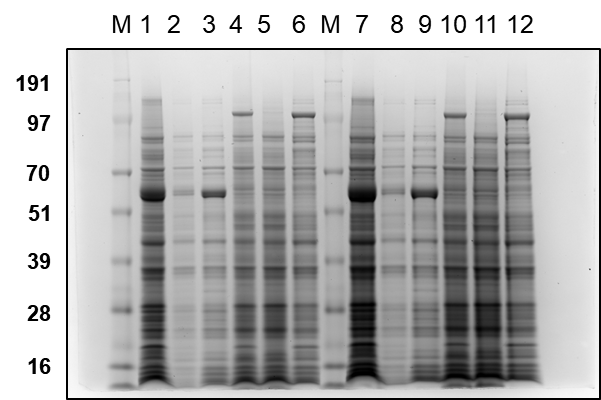


**Figure S3**. Uncropped version of the image used in Figure 1D. Solubility analysis of the protein particles. Information of lanes (1-6) is the same as described in the legend of Figure 1D. Lane 7-12 contain exactly the same sample as in lane 1-6 but double the volume of 10 µL.


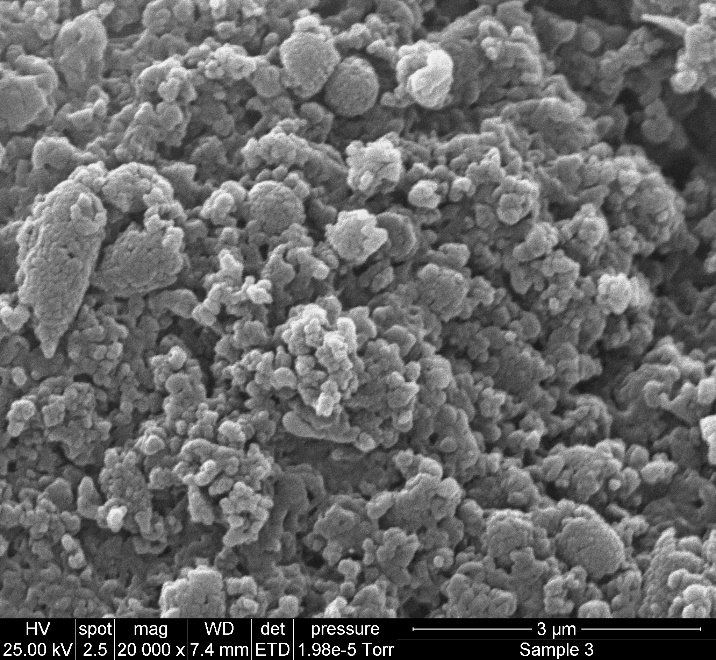


**A**

**B**


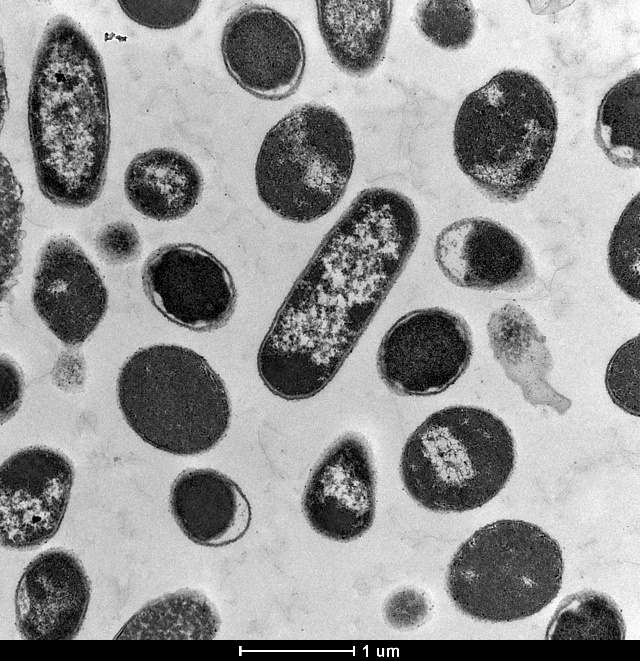


**Figure S4:** Uncropped version of the images used in Figure 1E. Electron microscopy analysis of protein particles. A; TEM, and B; SEM images of *E. coli* cells producing protein particles.

**Figure S5.** Assessment of MAP fusion antigen display on the surface of protein particles by ELISA. ELISA plates were coated with protein particles, ranging from 2 to 500 µg of protein per well and then probed with rabbit anti-rMAP polyclonal antibody. Bound antibody was detected with HRP-conjugated secondary antibody. Black circle (●) represents binding of antibody MAP fusion-PhaC protein particles and clear circle (🞅) represents binding of antibody to control protein particles.


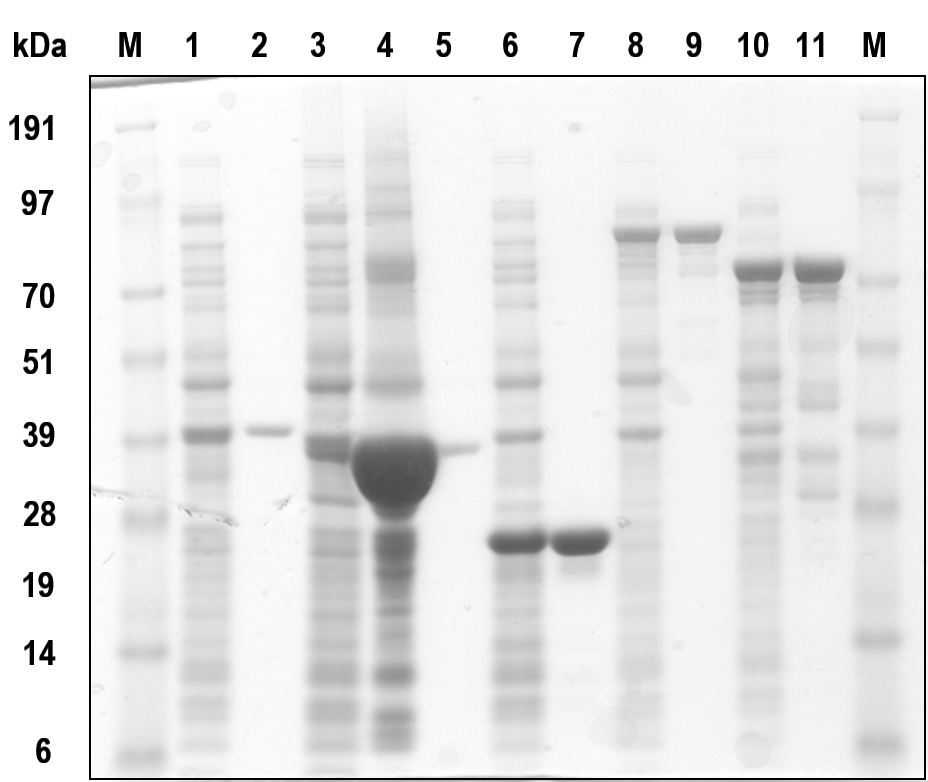


**Figure S6.** SDS-PAGE analysis of recombinant proteins produced in *E. coli*. Proteins in whole cell lysate and the purified recombinant proteins were separated by SDS-PAGE and stained with SimplyBlue. Lane M; SeeBlue Plus2 Pre-Stained Standard; lane 1, *E. coli* whole cell lysate of Ag85A; lane 2, purified Ag85A; lane 3 and 4, *E. coli* whole cell lysate of Ag85B; lane 5, purified Ag85B; lane 6, *E. coli* whole cell lysate of SOD; lane 7, purified SOD; lane 8-9, *E. coli* whole cell lysate of 74F and purified 74F (not used in the current study); lane 10, *E. coli* whole cell lysate of MAP fusion; and lane 11, purified MAP fusion.

**Table S1.** Liquid chromatography electrospray ionization tandem mass spectrometric analysis of MAP fusion antigens on protein particles

| **Protein sequence** | **Peptide fragments of various protein regions detected using LC/ESI-MS/MS** |
| --- | --- |
| MGPSLIGLAMGDAGGYKAADMWGPKEDPAWARNDPSLQVGKLVANNTRIWVYCGNGKPSDLGGDNLPAKFLEGFVRTSNLKFQDAYNGAGGHNAVWNFDANGTHDWPYWGAQLQAMKPDLQSVLGATPGAGPATAAATNAGNGQGTMAEYTLPDLDWDYAALEPHISGQINEIHHTKHHATYVKGVNDALAKLEEARANEDHAAIFLNEKNLAFHLGGMILAVNHPDQFIYAGSLSALLDPSQGMGPSLIGLAMGDAGGYKADAMWGPSSDPAWQRNDPSLHIPELVGHNTRLWVYCGNGTPSELGGANMPAEFLENFVRSSNLKFQDAYNAAGGHNAVFNFNANGTHSWEYWGAQLNAMKPDLQGTLGASPGGGGMLNQSVSATDTLTGAQENLGGLIQADAPIKPGDSGGPMVNSAGQVIGVDTAATDSYKMSGGQGFAIPIGRAMAVANQIRSGAGSNTVHIGPTAFLGLGVTDNNGNGARVQRVVNTGPAAAAGIAPGDVITGVDTVPINGATSMTEVLVMVGQVGPQVVNIDTKFGYNNAVGAGTGIVIDPNGVVLTNNHVISGATEISAFDVGNGQTYAVDVVGYDRTQDIAVLQLRGAAGLPTATIGGEATVGEPIVALGNVGGQGGTPNAVAGTSATGKGAAASTQEGKSQPFKVTPGPFDPATWLEWSRQWQGTEGNGHAAASGIPGLDALAGVKIAPAQLGDIQQRYMKDFSALWQAMAEGKAEATGPLHDRRFAGDAWRTNLPYRFAAAFYLLNARALTELADAVEADAKTRQRIRFAISQWVDAMSPANFLATNPEAQRLLIESGGESLRAGVRNMMEDLTRGKISQTDESAFEVGRNVAVTEGAVVFENEYFQLLQYKPLTDKVHARPLLMVPPCINKYYILDLQPESSLVRHVVEQGHTVFLVSWRNPDASMAGSTWDDYIEHAAIRAIEVARDISGQDKINVLGFCVGGTIVSTALAVLAARGEHPAASVTLLTTLLDFADTGILDVFVDEGHVQLREATLGGGAGAPCALLRGLELANTFSFLRPNDLVWNYVVDNYLKGNTPVPFDLLFWNGDATNLPGPWYCWYLRHTYLQNELKVPGKLTVCGVPVDLASIDVPTYIYGSREDHIVPWTAAYASTALLANKLRFVLGASGHIAGVINPPAKNKRSHWTNDALPESPQQWLAGAIEHHGSWWPDWTAWLAGQAGAKRAAPANYGNARYRAIEPAPGRYVKAKA | **Ag85A:** M1-R76, F82-V95  **SOD:** I166-K210  **Ag85B:** M245-R292, M310-R320, F326-N337  **74F:** A403-R484, V526-K539, A585-R593, I597-R603, G627-G641  **PhaC:** T642-K657, P670-K732, F744-R750, F757-R783, F788-R822, N827-R849, V877-K891, H906-R941, V962-R977, E1013-L1026, G1029-F1036, V1049-K1055, H1085-K1097, E1121-K1160, R1205-R1225 |

**Table S2:** Sequences of peptides used to stimulate axillary lymph node cells and splenocytes *in vitro*.

| **Number** | **Peptide ID** | **Sequence (N' - C')** |
| --- | --- | --- |
| 1 | MAP-Ag85A, Pep-1 | DQFVYAGSLSALLDS |
| 2 | MAP-Ag85A, Pep-2 | ELPQYLSAQKQVKPT |
| 3 | MAP-Ag85A, Pep-3 | RPGLPVEYLQVPSAA |
| 4 | MAP-Ag85A, Pep-4 | GDNLPAKFLEGFVRT |
| 5 | MAP-Ag85A, Pep-5 | AFEWYNQSGISVAMP |
| 6 | MAP-Ag85A, Pep-6 | MGPSLIGLAMGDAGG |
| 7 | MAP-Ag85A, Pep-7 | NDPSLQVGKLVANNT |
| 8 | MAP-Ag85A, Pep-8 | GAQLQAMKPDLQSVL |
| 9 | MAP-Ag85B, Pep-1 | FEWYYQSGLSVIMPV |
| 10 | MAP-Ag85B, Pep-2 | ELPSYLASNKGVKRT |
| 11 | MAP-Ag85B, Pep-3 | DQFIYAGSLSALLDP |
| 12 | MAP-Ag85B, Pep-4 | GANMPAEFLENFVRS |
| 13 | MAP-Ag85B, Pep-5 | AFSRPGLPVEYLQVP |
| 14 | MAP-Ag85B, Pep-6 | DAMWGPSSDPAWQRN |
| 15 | MAP-SOD, Pep-1 | AVLGYDTVGSRLLTF |
| 16 | MAP-SOD, Pep-2 | LDWDYAALEPHISGQ |
| 17 | MAP-SOD, Pep-3 | HHATYVKGVNDALAK |
| 18 | MAP-SOD, Pep-4 | TFQLYDQQANVPLGI |
| 19 | MAP-SOD, Pep-5 | VKADYVKAFWNVVNW |
| 20 | MAP-SOD, Pep-6 | VQKRYAAATSKAQGL |
| 21 | MAP-74F, Pep-1 | AAASYSTVLSGLTAG |
| 22 | MAP-74F, Pep-2 | LAYLYQTFMTNLFNT |
| 23 | MAP-74F, Pep-3 | GRMYSGPGAGSFVAA |
| 24 | MAP-74F, Pep-4 | LKFAPPIGYGALPKD |
| 25 | MAP-74F, Pep-5 | APASAAPSGLALDRF |
| 26 | MAP-74F, Pep-6 | YTSMYNAVKVPLGLT |
| 27 | MAP-74F, Pep-7 | APGDVITGVDTVPIN |
| 28 | MAP-74F, Pep-8 | GYNNAVGAGTGIVID |
| 29 | MAP-74F, Pep-9 | TNNHVISGATEISAF |
| 30 | MAP-74F, Pep-10 | DVVGYDRTQDIAVLQ |
| 31 | MAP-74F, Pep-11 | ENLGGLIQADAPIKP |
| 32 | MAP-74F, Pep-12 | VNSAGQVIGVDTAAT |
| 33 | MAP-74F, Pep-13 | SNTVHIGPTAFLGLG |
